# Events Across the Life Course Contribute to Higher Mobility Impairment Rates in Rural U.S.

**DOI:** 10.3389/fresc.2022.863716

**Published:** 2022-05-31

**Authors:** Catherine Ipsen, Bryce Ward, Andrew Myers

**Affiliations:** Rural Institute for Inclusive Communities, University of Montana, Missoula, MT, United States

**Keywords:** disability, injury, insurance, life-course model, rural

## Abstract

**Purpose:**

This paper investigates how life events such as injuries, health insurance coverage, geography, and occupation contribute to mobility disability rates over time. Findings can inform policies and practices to address factors that may contribute to disability in rural and urban areas.

**Methods:**

We utilized 27 waves of the National Longitudinal Survey of Youth (NLSY) data from 1979 to 2016 to explore how past injury, occupation, health insurance coverage, and rurality predicted mobility impairment at ages 40 and 50 using regression analysis.

**Findings:**

Rural respondents reported significantly higher rates of mobility impairment at age 40 and age 50 relative to people living in urban areas, and were more likely to report injury, work in high exertion occupations, and experience several pain-related health conditions. Using logistic regression and controlling for race and education, we found that people had higher odds of experiencing mobility impairment at age 40 if they reported a broken bone in the last 10 years, reported ever being knocked unconscious, had any workplace injury from 1988 to 2000, or lived in a rural area. People reported lower odds of mobility impairment if they had more consistent health insurance coverage over time. Further analysis showed that people consistently uninsured over time were 91% more likely to report mobility impairment at age 40 than those consistently insured.

**Conclusion:**

A better understanding of environmental factors associated with disability such as access to insurance, risk exposures, resources, and other place-based behaviors can inform additional strategies for reducing the severity and duration of mobility disability.

People in rural areas of the United States (US) are more likely to experience disability (1, 2). For example, 16.6% of residents in nonmetropolitan counties reported a disability compared to 12% of residents in metropolitan counties (3). This difference appears to persist across the life span as rural residents across all age cohorts report disability at similar rates as urban residents who are, on average, 10 years older (4).

While disability rates are typically higher in rural US counties across all disability types and age cohorts, mobility disability is the most prevalent (3, 4). Approximately 9% of the rural US population reports serious difficulty walking or climbing stairs compared to 6% of the urban population (3). Mobility impairment is associated with economic and social impacts for individuals, families, and communities (5, 6). For example, people who report mobility impairment at age 40 work approximately half as many hours over the next decade as individuals who do not report mobility impairment (B. Ward, unpublished data, 2020).

In part, mobility disability is related to life events, such as prior injuries and lack of access to appropriate health care at the time of those injuries, which may be more common in rural areas. For instance, rural areas in the US tend to have more physically demanding jobs associated with workplace injuries (7), and rural individuals have lower rates of insurance coverage and less access to specialty medical care for addressing health issues as they arise (8–10). A better understanding of how life events shape future outcomes can help identify appropriate interventions, services, and accommodations in the workplace and in healthcare delivery.

We used data from the National Longitudinal Survey of Youth 1979 to explore how life events (i.e. injuries, health insurance coverage, geography, and high risk occupations) contribute to higher rates of mobility disability over time. Knowledge about specific upstream factors that contribute to downstream mobility disability can inform policies and practices that may mitigate the incidence and impacts of life events and promote overall wellbeing.

## The Life Course Model

The ecological model of disability describes disability as the outcome of dynamic interactions between a person and their environment (11, 12). The life course model can be used to operationalize the ecological model of disability by tracking individual and environmental interactions over time to predict downstream outcomes, such as chronic disease and disability.

Rather than focus on current conditions to explain health outcomes, the life course model attempts to understand how exposures or risks at different life stages influence health outcomes later in life. The life course model also explores how cumulative interactions between an individual and contextual settings influence the likelihood of developing non-communicable disease or disability (e.g., chronic pain, obesity, diabetes, cardiovascular disease, cancer, chronic respiratory disease, musculoskeletal disorders, and depression).

Within the life course model, Merlo highlights that those with similar socioeconomic factors experience similar outcomes (13). Geographic units of analysis such as neighborhoods, cities, or counties can be used to understand common or associated risk factors across different communities. For example, risk or exposure in a high resourced setting may result in a short-term or temporary impact, e.g., transitory disability (14), whereas the same risk or exposure in a low resourced setting might result in a precipitous decline in health or death (15). In other words, community context matters.

A simple life-course model of disability suggests at least three possibilities to explain persistent rural and urban differences in mobility disability rates (16–19). First, people in rural areas are more likely to acquire disability through injury or illness. This includes introduced risk from both behavioral norms and employment settings. For instance, people in rural areas self-report lower rates of seatbelt use, which translates into increased risk of car accident-related morbidity and mortality (20). Likewise, high-risk for injury occupations such as mining, logging, agriculture, and manufacturing are more prevalent in rural, relative to urban areas (7).

Second, people in rural areas do not achieve the same recovery from injury or illness as their urban counterparts due to compromised access to, or use of, health care. This can stem from a variety of factors including availability of specialized care, insurance coverage, and other community based psychosocial factors. It is well-documented that rural communities have lower rates of per capita specialty care and insurance coverage (8, 10). Additionally, they appear to utilize health care differently, which may introduce additional factors impacting disability outcome. For instance, Young et al., found significant rural-urban differences in worker's compensation healthcare claims after controlling for demographics, injury type, and severity (21). Specifically, rural workers used significantly fewer physical therapy services than their urban counterparts. Lower rates of health care utilization also resulted in different work disability durations for rural workers based on severity of injury. For more severe injuries, rural workers experienced longer work disability durations than urban workers. Conversely, for less severe injuries, rural workers had shorter work disability durations. Within the life course model, it is possible that lower rates of physical therapy services had direct impacts - longer duration of work disability for severe injury, and indirect impacts - incomplete recovery leading to higher rates of mobility impairment over time (21).

Finally, environmental factors in rural areas can impact disability outcomes as well. For example, rural communities typically have fewer employment choices that can accommodate functional limitations associated with disability (22). Inaccessible community infrastructure such as lack of sidewalks, limited public transportation, crosswalks without audio signals, or inaccessible buildings can create barriers to social participation and medical services (23–25). Fewer supports and lack of accessibility can introduce additional socioeconomic risks, such as declining wages, lost employment, and social isolation (26).

In this paper, we explore how life events such as occupation, injury, access to insurance, and geography predict disability status later in life. The findings make an important contribution to the field due to the longitudinal nature of the NYLS79 data and ability to explore cause and effects over time. While we know that rural people experience different rates of disability and health conditions, exploring the precursors to each over time provides a more nuanced understanding for addressing policies and practices that may improve quality of life.

## Methods

### National Longitudinal Survey of Youth 1979 (NLSY79)

The NLSY79 is administered by the Bureau of Labor Statistics (BLS) to explore educational, labor force, and family experiences across the life span. The NLSY79 consists of a nationally representative sample of 12,686 US residents born between 1957 and 1964. Respondents completed their first wave of data in 1979, when they were between the ages of 14 and 22. Since that time, a subset of respondents have been resurveyed at multiple times to explore a range of life transitions related to education, residence, employment, income, family composition, and health (27). The NLSY79 was conducted annually from 1979 to 1994, and then bi-annually from 1994 forward. Some survey modules were asked consistently over time, while others were asked at specific points in time, such as when the respondent turned a specific age (e.g., 40+ and 50+ interviews).

### Measures

For our analyses, we utilized 27 waves of data spanning 37 years between 1979 and 2016. Due to the length and complexity of the NLSY79 data, we only describe variables used in our analyses and how they were constructed. Several measures were calculated using data from the 40+ health interviews collected during the first survey administration after the respondent turned 40 years old. Other variables were drawn from multiple waves of data to construct proxy measures about experiences and conditions prior to age 40.

#### Disability

We used dichotomized variables to indicate mobility disability at age 40 and 50. Respondents were classified as having a mobility disability if they answered yes to “having a lot or a little trouble climbing several flights of stairs,” as reported in the 40+ and 50+ health interviews.

#### Occupational Exertion by Age 40

We calculated a proxy variable for share of work history engaged in high exertion occupations by age 40. At each wave of data collection, the NLSY79 collects work history data since the preceding interview including time spent working in different occupations. We recoded NLSY79 occupation codes from different years into a consistent occupational coding scheme based on Pollard (28). Next, we identified high-physical exertion occupations based on questions included in two waves of data collection (1998 and 2000), which asked respondents two indicators of work intensity: (1) Does your job require lots of physical effort? [all, most, some, or none of the time], and (2) My job requires lifting heavy loads, stooping, kneeling, crouching, walking or other types of physical effort [rarely, a little, occasionally, most of the time]. We classified an occupation as high intensity if over 50% of respondents in that job category reported the job required high levels of physical exertion (i.e., reported physical effort all or most of the time AND required lifting etc. occasionally or most of the time). Based on this information, we computed the total time individuals spent in high exertion occupations (average weekly hours ^*^ number of weeks), divided by total number of hours worked to arrive at a share of time working in high exertion occupations. When weeks or hours were coded using a range, we used the low end of the range. This constructed variable ranged from 0 (never worked in a high-exertion occupation) to 1 (always worked in high-exertion occupation).

#### Injury

We used three variables to estimate injury, including broken bones, concussion, and workplace injury. The first two variables came from the 40+ interview, where respondents indicated if (1) they had broken a bone in the last 10 years, and (2) if they had ever been knocked unconscious. The workplace injury variable was derived from 9 waves of NLSY79 data representing 12 years (1988-2000) when respondents indicated if they had any workplace injury since their last NLSY79 survey. We used these data to create a binary variable equal to one if the respondent reported any workplace injury and zero otherwise.

#### Work Limitation

Each wave of the NLSY79 asks respondents whether a health condition makes them unable to work, limits the amount they work, and/or limits the type of work they can do. For each wave of data, respondents who answered yes to one or more of these questions were assumed to have a work disability. We created an indicator equal to one if a respondent reported a work limitation in any wave through the 40+ health interviews

#### Heath Indicators

The NLSY79 asked respondents about self-reported health problems and diagnosed health conditions at the 40+ health interview. *Self-reported* health problems included responses to the question “do you have any of the following health problems” and included an exhaustive list of conditions including joint pain and stiffness; asthma; back pain; problems with feet and legs; kidney or bladder problems; stomach or intestinal ulcers; high cholesterol; chest pain or abnormalities; low blood pressure; sinus problems or allergies; frequent indigestion or intestinal troubles; depression or anxiety; painful joints or bursitis; lameness or paralysis; trick or frozen shoulder, knee, or elbow; tuberculosis, jaundice or hepatitis; headaches, dizziness or fainting; eye trouble; ear nose and throat trouble; tooth and gum trouble; skin diseases; thyroid trouble; tumors or growths; deformities; loss of finger or toe; neuritis or nerve dysfunction; epilepsy; frequent trouble sleeping; frequent urinary tract infection; osteoporosis; hardening of the arteries; and anemia. *Diagnosed* health conditions were phrased “Has a doctor ever told you that you have X” and included high blood pressure or hypertension; diabetes; cancers; chronic lung disease, chronic bronchitis, or emphysema; heart attack, coronary heart disease, angina, congestive heart failure, or other heart problems; stroke; emotional, nervous or psychiatric problems; and arthritis or rheumatism.

#### Health Insurance

We utilized a proxy to estimate health care access over time. Starting in 1989, and in subsequent waves, the NLSY79 surveys asked respondents if they had insurance currently. We calculated the share of “yes” responses across time periods to construct a variable capturing the share of time with health insurance up to age 40. Scores ranged from 0 (no insurance at any time period) to 1 (current insurance at every time period).

#### Rural Environment

Respondents were classified as living in rural or urban locations based on NLSY79 calculated urban-rural variables derived from Metropolitan Statistical Area (MSA) geo-codes. We defined residents as rural if they lived in a nonmetro area and urban if they lived in a metro area when they responded to the 40+ interviews. We also calculated the duration of rural residence, based on the share of data waves where the respondent was classified as living in rural areas by the 40+ interview. The average person living in a rural area at age 40 reported living in a rural area during 73% of their NLSY interviews between 1979 and 2016, while the average urban resident reported living in a rural area in 10% of their NLSY interviews. From this information, we determined that a dichotomous variable was appropriate for our analyses.

### Participants

Our sample was limited to 8,451 respondents who responded to the mobility disability question “Do you have trouble climbing several flights of stairs?”, and 13.7% reported mobility impairment at the 40+ survey. Participants were roughly split between male and females (51 vs 49%). Of these 30.7% identified as Black, 19.6% identified as Hispanic, and 49.7% identified as some other race (i.e., not Black, not Hispanic). Approximately 14% of the sample were classified as living in a rural location at the 40+ interview. People who reported living in a rural location at age 40 were significantly more likely to be non-black, non-Hispanic (47.4% urban vs. 65.2%).

### Data Analyses

We downloaded NLSY79 data files into STATA V. 16 to construct case files and create model variables. We uploaded model variables into SPSS V. 25 to conduct analyses. Our analytical approach first explored differences in mobility impairment, injury, occupational exertion, health insurance coverage, and health conditions between people who lived in urban and rural locations. Then, we used logistic regression to explore how past injury, health insurance coverage, and rural residence predicted mobility impairment, after controlling for race and education.

## Results

### Urban and Rural Comparisons of Life Events

Table 1 compares rural and urban rates of mobility impairment, and several explanatory variables including socio-demographics, injuries, workplace demands, and health insurance. Rural respondents were significantly more likely to report mobility impairment at ages 40 and 50, ever being unconscious, and ever suffering a workplace injury. They also reported a significantly smaller share of time with health insurance coverage. For instance, people who lived in urban areas reported health insurance coverage in 81 percent of their interviews through age 40, while people in rural areas reported health insurance coverage in 77 percent of interviews.

**Table 1:** Urban and rural *t*-test comparisons of socio-demographics, mobility impairment, injury, work history, and insurance.

	**Urban % or *M***	**Rural** **% or *M***	***p***
Female	51.1%	50.0%	0.300
Education–high school graduate	42.2%	50.1%	^***^
Education–some college of more	48.7%	36.8%	^***^
White–not hispanic	47.4%	65.2%	^***^
A little or a lot of difficulty climbing stairs (age 40)	13.2%	17.3%	^***^
A little or a lot of difficulty climbing stairs (age 50)	24.0%	28.0%	0.004^**^
Broken bone in last 10 years (through age 40)	12.9%	14.9%	0.056
Ever unconscious (through age 40)	8.2%	10.2%	0.022^*^
Any workplace injury (1988–2000)	32.1%	35.3%	0.032^*^
Ever reported physical limitation restricts amount or type of work (through age 40)	43.2%	45.9%	0.073
Mean share of work-life in high physical exertion occupations (through age 40)	23.3	32.1	^***^
Mean share of observations with health insurance (through age 40)	80.6	76.7	^***^

*Analyses of age 40 outcomes include 8,451 observations. Analyses through age 50 include 7,588 observations*.

* *p < 0.05*,

** *p < 0.01*,

*** *p < 0.001*.

People in rural areas also spent significantly more time working in high exertion occupations. We explored how this might impact the probability of workplace injuries and found that a one standard deviation increase in share of employment in a high exertion occupation increased the odds of workplace injuries by 31%, using bivariate logistic regression.

### Illness and Health Conditions

Like disability, life-events may shape the development of health conditions. Table 2 includes comparisons of health conditions between people living in rural and urban locations and for individuals with and without mobility impairment at age 40. Relative to urban people, respondents living in rural areas were more likely to report conditions like back problems, joint pain, trouble sleeping, and arthritis/rheumatism, which are often associated with more physically demanding employment (7). Comparatively, many conditions were not more common in rural areas, such as cancer, diabetes, asthma, chronic lung disease, anemia, or epilepsy. People with mobility impairment at age 40 reported significantly higher rates of every reported health condition than individuals who did not report mobility impairment.

**Table 2:** Health problem comparisons at age 40 (*n* = 8451).

	**Urban %**	**Rural %**	***p***	**No** **Disability %**	**Has Disability %**	***p***
Back problems	23.9	29.7	^***^	20.4	52.3	^***^
Joint pain/frequent leg cramps/bursitis	14.2	18.8	^***^	10.1	44.3	^***^
Frequent trouble sleeping	15.5	20	^***^	12.2	41.2	^***^
Ever had arthritis or rheumatism	11.2	14.8	^***^	8.1	34.1	^***^
Indigestion/intestinal/gall bladder problems	8.7	11.8	0.001^**^	7.1	22.3	^***^
Chest pain/pounding heart/other heart problems	5.4	7.9	0.001^**^	4	17.4	^***^
Depression/excess worry/nervous problems	12.7	15.9	0.003^**^	9.8	34.2	^***^
Diagnosed hypertension	16.7	20.3	0.003^**^	14.8	32.5	^***^
Frequent headaches/dizzy/fainting	10.5	13.3	0.004^**^	8.5	26.7	^***^
Ulcer	2.5	3.9	0.005^**^	1.9	7.9	^***^
Foot and leg problems	19.5	22.5	0.015^*^	14.1	56.7	^***^
Diagnosed stoke	0.8	1.3	0.047^*^	0.5	3.3	^***^
Kidney or bladder problems	4.5	5.7	0.063	3.4	12.8	^***^
Lameness/paralysis/polio	1.1	1.7	0.065	0.5	5.8	^***^
Diagnosed emotional/nervous problems	7.3	8.7	0.085	5.3	20.5	^***^
Severe tooth or gum trouble	6.1	7.4	0.1	5	13.9	^***^
Stomach or intestinal problems	5.2	6.3	0.106	3.9	14.4	^***^
Frequent colds/sinus/allergies	23.9	25.8	0.155	21.9	38.2	^***^
Diagnosed heart problems	2.8	3.5	0.194	1.9	9.1	^***^
Diagnosed chronic lung disease	2.8	3.5	0.206	1.7	10	^***^
Hardening of the arteries	0.3	0.5	0.213	0.3	0.6	0.049^*^
Osteoporosis	0.9	1.2	0.216	0.5	3.2	^***^
Diagnosed congestive heart failure	0.3	0.5	0.216	0.1	1.6	^***^
Neuritis	0.5	0.7	0.23	0.3	1.6	^***^
Frequent urinary tract infections	2.1	1.7	0.301	1.6	4.9	^***^
Skin diseases	2.7	3.2	0.304	2.4	5.1	^***^
Ear, nose, or throat problems	6.1	6.8	0.333	4.9	14.1	^***^
Scarlet/rheumatic fever, TB, jaundice, hepatitis	1.6	2	0.359	1.3	4.2	^***^
Diagnosed non-skin cancer	2.1	1.7	0.432	1.6	5.1	^***^
Thyroid trouble or goiter	3.7	3.3	0.47	3.1	6.7	^***^
Diagnosed diabetes	5.5	5	0.485	4.1	13.3	^***^
Eye trouble (not glasses)	5.2	4.8	0.553	3.8	14.2	^***^
Asthma	8	7.5	0.555	5.9	20.7	^***^
Anemia	5.3	4.9	0.564	4.1	12.1	^***^
Epilepsy or fits	1.1	1.2	0.66	0.8	3	^***^
High cholesterol	10.8	11.2	0.698	9.8	18.1	^***^
Low blood pressure	5.3	5.5	0.781	4.6	9.6	^***^

* *p < 0.05,*

** *p < 0.01,*

*** *p < 0.001*.

### Predicting Mobility Impairment With Logistic Regression

Table 3 shows a logistic regression predicting mobility impairment at age 40. After controlling for race/ethnicity and educational attainment, explanatory variables included indicators of past injury (i.e., broken bone in the last 10 years, ever unconscious, and any workplace injury), share of work-life in high exertion occupations, share of observations with health insurance, and living in rural at age 40. We checked model variables for potential multicollinearity and found none.

**Table 3:** Logistic regression on mobility impairment at age 40 (*n* = 8,451).

	**B**	**SE**	**p**	**OR**	**Lower**	**Upper**
Not black, not Hispanic	−0.405	0.069	0.000	0.667	0.583	0.763
Education (Ref: no Ged)						
High school graduate	−0.445	0.100	0.000	0.641	0.527	0.78
Some college or more	−0.813	0.113	0.000	0.444	0.355	0.554
Broken bone in last 10 years at age 40	0.629	0.084	0.000	1.876	1.591	2.213
Ever unconscious by age 40	0.396	0.104	0.000	1.486	1.212	1.822
Any workplace injury by age 40	0.347	0.067	0.000	1.415	1.24	1.615
Share of worklife in high exertion occupation at age 40	−0.15	0.125	0.233	0.861	0.673	1.101
Share of health insurance up to age 40	−0.315	0.119	0.008	0.73	0.578	0.922
Rural residence at age 40	0.315	0.087	0.000	1.371	1.156	1.626
Constant	−1.22	0.134	0.000	0.322		

*Nagelkerke R2 = 0.055*.

People who reported a broken bone, being knocked unconscious, or having workplace injury were 87, 48, and 41% more likely to report mobility impairment at age 40, respectively. After controlling for these injuries, the share working in high exertion occupations was not a significant predictor. Living in a rural location at age 40 increased the odds of mobility impairment by 37%, while share of health insurance coverage lowered the odds by 73%. Further analysis with bivariate logistic regression showed that people consistently uninsured over time were 91% more likely to report mobility impairment at age 40 than those consistently insured.

Table 4 shows the relationship between life course indicators by age 40 and reported impairment at age 50. Of the sample completing the NLSY at age 50 (*n* = 7,588), 15.8% became impaired between 40 and 50 (transitioned into disability), 4.6% reported impairment at 40 but not at 50 (transitioned out of disability), and 8.7% reported impairment at 40 and 50 (enduring disability). We report the odds of having a mobility impairment at age 50 for each of these groups, using the same explanatory variables as reported for mobility impairment at age 40.

**Table 4:** Logistic regression models on mobility impairment at age 50.

	**Model 1Δ** **(*****n*** **= 6575)**		**Model 2 +** **(*****n*** **= 1158)**		**Model 3 □** **(*****n*** **= 7588)**	
	**OR**	**Sig**	**OR**	**Sig**	**OR**	**Sig**
Not black, not hispanic	0.677	0.000	0.794	0.097	0.809	0.015
Education (Ref: no Ged)						
High school graduate	0.742	0.006	1.165	0.447	0.623	0.000
Some college or more	0.545	0.000	1.363	0.163	0.456	0.000
Broken bone in last 10 years at age 40	1.277	0.012	0.687	0.033	1.697	0.000
Ever unconscious by age 40	0.940	0.613	0.684	0.088	1.423	0.007
Any workplace injury by age 40	1.214	0.005	0.941	0.655	1.453	0.000
Share of worklife in high exertion occupation at age 40	1.123	0.361	0.796	0.350	0.900	0.511
Share of health insurance up to age 40	0.568	0.000	1.741	0.030	0.573	0.000
Rural residence at age 40	1.147	0.140	1.032	0.858	1.339	0.008
Constant	0.545	0.000	0.311	0.000	0.214	0.000
Nagelkerke R^2^	0.043		0.033		0.045	

*ΔModel 1 predicts people who transitioned into disability - did not report impairment at age 40 but did report impairment at age 50 (1,196 out of 6,575 possible)*.

*+Model 2 predicts people who transitioned out of disability - reported impairment at age 40 but did not report impairment at age 50 (346 out of 1,158 possible)*.

*□Model 3 predicts people with enduring disability - reported impairment at age 40 and age 50 (667 out of 7,588 possible)*.

#### Transitioned Into Disability Between 40 and 50

The first results column shows how conditions at age 40 predict who will become impaired between ages 40 and 50. The logistic regression was confined to the 6,575 people who did not have a mobility impairment at age 40. Evidence of injury increased the odds of becoming impaired by age 50, while more consistent health insurance coverage lowered the odds

#### Transitioned out of Disability Between 40 and 50

The second results column reports on those who reported mobility impairment at age 40 but did not report impairment at age 50. This logistic regression was confined to the portion of the sample who had impairment at age 40 (*n* = 1,158). In this model, predictors worked in the opposite direction, where reporting a broken bone at age 40 lowered the odds of not reporting mobility impairment at age 50 and having a higher share of insurance coverage at age 40 increased the odds of not reporting mobility impairment at age 50.

#### Reported Enduring Disability at Ages 40 and 50

The final column focused on the people who reported mobility impairment at both age 40 and age 50 (i.e., enduring disability). Conditions at age 40 strongly predicted consistent impairment. People who reported a broken bone or having workplace injury at age 40 were 69 and 45% more likely to report mobility impairment at age 50. Similarly, consistently having health insurance was associated with a 75% percent decrease in the odds of reporting consistent impairment.

## Discussion

A better understanding of disability and its precursors informs strategies for future interventions, and provides guidance for allocating health resources to those who may be more likely to experience disability across the lifespan. Data from the present study highlight contextual factors that may play a role in disability severity and duration, and how these factors vary across urban and rural locations. Specifically, rural respondents by age 40 had significantly higher odds of having a broken bone, concussion, or workplace injury in the prior 10 years than respondents from metro locations. Controlling for rurality revealed that these types of injuries were also significant factors on their own.

Many injuries occurred at the workplace and led to higher odds of mobility disability. This highlights the importance of workplace safety and safety culture to reduce accidents and the importance of effective medical care when injuries occur. Because workplace injuries did not happen uniformly across geography, it suggests rural and urban differences in injury treatment (21). One strategy to address this is to increase worker's compensation benefits immediately following injury (21, 29). Worker's compensation benefits typically cover medical costs to treat injury, temporary disability benefits to offset lost wages, and permanent disability benefits when workplace injuries lead to permanent impairment (30). Temporary disability benefits vary across states, but typically pay a portion of lost wages (e.g., 2/3 of wages) after a specified waiting period (e.g., one-week). Because people in rural areas experience higher rates of poverty, lower wages, and fewer employment options, this may shape decision making to seek care and access temporary disability benefits (31). Providing more liberal payments for lost wages and removing waiting periods, may increase the probability of a more complete recovery (32).

Further analysis of the data showed that some injuries were independent of the workplace (i.e., not reporting workplace injury, but reporting broken bones and/or concussion). Different rural and urban prevalence rates may point to variations in behavioral norms and activities, including decision-making to seek care. We know that the rate of enduring disability (i.e., reporting disability at both age 40 and age 50) was lower for respondents reporting more instances of health care insurance coverage up to age 40. This evidence suggests that access to insurance is a particularly important factor for adequately addressing injury and lowering the odds of experiencing long-term disability and highlights the value of programs such as Medicaid expansion for the uninsured (33).

Other care-seeking factors are at play in rural communities. For instance, rural people have more limited access to specialty healthcare services, must travel further to access services, or may have privacy concerns related to healthcare visits (34). Policies and infrastructure to increase access to telehealth may be one strategy to reduce these types of care-seeking barriers. As telehealth access increases, additional variables that account for access to medical services and health seeking behaviors may provide additional information for understanding these impacts.

Onset of disability is associated with economic costs for individuals and families (5). For instance, a study using data from the Panel Study of Income Dynamics (PSID), reported that those with onset of chronic or severe disability between the ages of 18 and 65 experienced a 79% reduction in earnings and a 22% decline in food consumption 10 years later. However, this same study showed that negative economic outcomes were significantly moderated 10 years after onset for respondents with chronic but not severe, temporary, and one-time only reported disability (35). This suggests that interventions that can improve access for people with disabilities early-on (i.e., healthcare, Medicaid, workplace accommodations) may improve long-term economic outcomes.

These differences highlight the importance of addressing the onset of disability with appropriate medical access, behavioral, and social/community interventions. Looking at data using a life course model allows us to see how severe or chronic disability unfolds over time and offers opportunities to address risk and exposure incidents that reduce the incidence and severity of long-term disability. Additional research based on the life-course model could focus on additional risk and protective factors, such as adverse childhood events or childhood access to consistent healthcare.

## Limitations

This paper and analyses were limited by the NLSY79 survey questions. First, the NLSY79 does not measure the varied experience of disability. Our analyses were focused on respondents reporting mobility impairment, defined as having a lot or a little trouble climbing several flights of stairs at the 40+ and 50+ health interviews. Disability rates, however, are higher in rural areas for multiple disability types, including hearing, vision, cognitive, ambulatory, self-care, and independent living disabilities (36). Similarly, many explanatory variables were proxies which may have under or over-estimated specific characteristics, such as health insurance coverage, duration of disability, and rural status. Despite imprecise measurement, however, the models provided consistent evidence about the relationships between environmental factors and subsequent disability experience.

## Conclusions

The ecological model of disability posits that disability is the result of personal and environmental factors. The life-course model expands on this theory by highlighting how personal/environmental interactions across the life-course factor into the longer-term experience of disability. Better understanding of environmental factors such as access to insurance, risk exposures, resources, and other place-based behaviors inform additional strategies for reducing the severity or duration of disability.

## Data Availability Statement

Publicly available datasets were analyzed in this study. This data can be found here: https://www.nlsinfo.org/content/cohorts/nlsy79.

## Author Contributions

BW, CI, and AM developed the presented idea, discussed the results, and contributed to the final manuscript. BW and CI developed the theory and computations. CI and AM verified the analytical methods. All authors contributed to the article and approved the submitted version.

## Funding

This work was supported by the Research and Training Center on Disability in Rural Communities (RTC:Rural) under a grant from the National Institute on Disability, Independent Living, and Rehabilitation Research (NIDILRR; Grant number 90RTCP0002). NIDILRR is a Center within the Administration for Community Living (ACL), Department of Health and Human Services (HHS). The research does not necessarily represent the policy of NIDILRR, ACL, or HHS and one should not assume endorsement by the federal government.

## Conflict of Interest

The authors declare that the research was conducted in the absence of any commercial or financial relationships that could be construed as a potential conflict of interest.

## Publisher's Note

All claims expressed in this article are solely those of the authors and do not necessarily represent those of their affiliated organizations, or those of the publisher, the editors and the reviewers. Any product that may be evaluated in this article, or claim that may be made by its manufacturer, is not guaranteed or endorsed by the publisher.
